# Auricular neural stimulation as a new non-invasive treatment for opioid detoxification

**DOI:** 10.1186/s42234-020-00044-6

**Published:** 2020-03-30

**Authors:** Imran S. Qureshi, Timir Datta-Chaudhuri, Kevin J. Tracey, Valentin A. Pavlov, Andrew C. H. Chen

**Affiliations:** 1 Department of Psychiatry, Zucker Hillside Hospital, Northwell Health, Glen Oaks, NY USA; 2 Chemical Dependency Dual Diagnosis Outpatient Facility, Department of Psychiatry, Staten Island University Hospital, Northwell Health, Staten Island, NY USA; 3 Zucker School of Medicine at Hofstra/Northwell, Hempstead, NY USA; 4 Institute of Bioelectronic Medicine, The Feinstein Institutes for Medical Research, Manhasset, NY USA

**Keywords:** Electrical nerve stimulation, Non-invasive intervention, COWS score, Addiction, Opioid epidemic, Auricular vagus nerve stimulation

## Abstract

The recent opioid crisis is one of the rising challenges in the history of modern health care. New and effective treatment modalities with less adverse effects to alleviate and manage this modern epidemic are critically needed. The FDA has recently approved two non-invasive electrical nerve stimulators for the adjunct treatment of symptoms of acute opioid withdrawal. These devices, placed behind the ear, stimulate certain cranial nerves with auricular projections. This neural stimulation reportedly generates a prompt effect in terms of alleviation of withdrawal symptoms resulting from acute discontinuation of opioid use. Current experimental evidence indicates that this type of non-invasive neural stimulation has excellent potential to supplement medication assisted treatment in opioid detoxification with lower side effects and increased adherence to treatment. Here, we review current findings supporting the use of non-invasive neural stimulation in detoxification from opioid use. We briefly outline the neurophysiology underlying this approach of auricular electrical neural stimulation and its role in enhancing medication assisted treatment in treating symptoms of opioid withdrawal. Considering the growing deleterious impact of addictive disorders on our society, further studies on this emerging treatment modality are warranted.

## Background

Approximately 36 million people are affected with opioid use disorder globally (Parrino et al. 2015). Opioid dependence is one of the emerging challenging public health issues across North America adversely impacting individual, social, and occupational functioning. Every day, more than 130 people in the United States alone die after overdosing on opioids (CDC/NCHS 2018). Recently, there has been a substantial increase in use and abuse of prescription opioids, illicit drugs, and synthetic opioids such as fentanyl (Parrino et al. 2015). The widespread availability of opioids and related medications coupled with inadequate education within the general population regarding its long-term consequences, and the potential for subsequent dependence, has immensely contributed to this epidemic. The misuse of and addiction to opioids, affects health as well as social and economic welfare; in 2013, the annual economic burden of opioid use disorder was nearly $80 billion, of which nearly $30 billion were due to healthcare costs (Florence et al. 2016).

Currently, there are three approved medications for the treatment of opioid addiction, including methadone, buprenorphine, and naltrexone (McElrath 2018; Maglione et al. 2018; Herring et al. 2019; Blanco and Volkow 2019) (Table 1). Buprenorphine, for example, has been quite effective in randomized clinical trials (Herring et al. 2019; Gowing et al. 2017) for managing acute symptoms during the detoxification and maintenance phases of opioid addiction treatment. However, despite the widespread availability of this medication, it is associated with significant undesirable symptoms of withdrawal during the initial course of treatment (Table 1). Naltrexone, similarly, helps maintenance of abstinence from opioids yet may worsen opioid withdrawal symptoms as naltrexone is an opioid antagonist. The Food and Drug Administration (FDA) recommends that individuals should be abstinent from opioid drugs for a week to 10 days before starting naltrexone (Blanco and Volkow 2019; Kleber 2007), leading to lower rates of naltrexone prescription for opioid abuse management.

**Table 1:** Currently available treatments for opioid withdrawal/opioid use disorder

Treatment	Primary Mechanism of Action	FDA Approval Status	Pros	Cons	References
Buprenorphine	Mu receptor partial agonist	Approved	1. Ceiling effect to prevent overdose. 2. Prevents euphoria due to partial agonism therapy. 3. Long-term injection formulation is available. 4. Naloxone conjunction prevents misuse.	1. Long term may lead to reduced libido. 2. May cause nausea and constipation. 3. Potential dependence on higher dosage.	(Gowing et al. 2017; Elkader and Sproule 2005)
Methadone	Mu receptor full agonist	Approved	1. Effective in resistant cases of opioid dependence. 2. Effective in HIV and HCV infectious states 3. Favorable prognostic outcome with history of non- compliance.	1. Close supervision for its administration is required. 2. More risk of sustaining dependence and GI related side effects. 3. Increased risk of overdose if taken in conjunction with other controlled sedatives.	(Mattick et al. 2008; Eap et al. 2002; Bell and Strang 2020)
Naltrexone	Mu receptor antagonist	Approved	1. Effective in co-existing alcohol and opioid dependence. 2. Prevents reinforcing effects of opioid use. 3. Long-term injection formulation is available.	1. Avoid in advanced liver diseases. 2. Caution in those who have underlying severe depressive illness.	(Blanco and Volkow 2019; Kleber 2007; Koehl et al. 2019)
Lofexidine	Alpha 2 receptor agonist	Approved	1. Effective in withdrawal state driven by high sympathetic flow. 2. Facilitates detoxification from opioids. 3. First FDA approved non-opioid medication.	1. May precipitate sedation, drowsiness. 2. Blood pressure monitoring required - Risk of inducing hypotension.	(McCambridge et al. 2007; Pergolizzi Jr. et al. 2019; Gerra et al. 2001; Kuszmaul et al. 2019)
Clonidine	Alpha 2 receptor agonist	Off label	1. Ameliorates withdrawal symptoms. 2. Helps in alleviating anxiety and restlessness associated with withdrawal.	1. Caution for sudden hypotension. 2. Precipitates sedation and dizziness	(Gerra et al. 2001; Kuszmaul et al. 2019)
Baclofen	GABA-B receptor agonist	Off label	1. Alleviates muscle spasms. 2. Facilitates detoxification course through muscular symptom neutralization	1. Abuse potential. 2. Sedations, drowsiness and dizziness as part of side effects	(Cousins et al. 2002; Rahimi-Movaghar et al. 2018)
Benzodiazepines	Increase effectiveness of GABA-A receptor complex	Off label	1. Rapid relief of symptoms	1. Highly addictive. 2. Severe side effects including respiratory depression.	(Stein et al. 2016; Lintzeris and Nielsen 2010)
Neural Stimulation	Stimulation of V, VII, IX, X cranial nerves	Approved	1. First FDA approved non systemic based device for alleviation of withdrawal symptoms. 2. Reduces use of medications during acute withdrawal. 3. Non-invasive, well tolerable, with no major side effects.	1. Cost-effectiveness unknown 2. Studies for long-term use are not available	(Miranda and Taca 2018; Baker and Chang 2016)

Although symptoms of opioid withdrawal generally resolve after 5–14 days (depending on the half-life of the opioid), the distress in the first few days after abrupt discontinuation can be rather severe. Without adequate treatment, many patients are unable to complete opioid discontinuation (Mattick et al. 2008; Kosten and Baxter 2019) as avoidance of symptoms of opioid withdrawal often becomes the most powerful force driving continued use (Cicero and Ellis 2017). Besides buprenorphine and methadone, some supportive pharmacotherapeutic agents are available to mitigate uncomfortable symptoms associated with acute phase treatment including clonidine, baclofen, ibuprofen and lofexidine (Kleber 2007; Diaper et al. 2014). However, the overall effectiveness of the supportive medications is not satisfactory (Hermann et al. 2005). Furthermore, some medications used to manage acute opioid withdrawal are a subject of serious concerns. Benzodiazepines, for instance, are a commonly used class of medications (Stein et al. 2016), yet they can be additive and contribute to severe respiratory suppression and other side effects (Lintzeris and Nielsen 2010). Therefore, development of more efficient and with less side effects, pharmacological approaches for the treatment of opioid use disorder remains ongoing. Simultaneously, novel non-pharmacological interventions that can assist in treatment should also be explored.

**Fig. 1:**
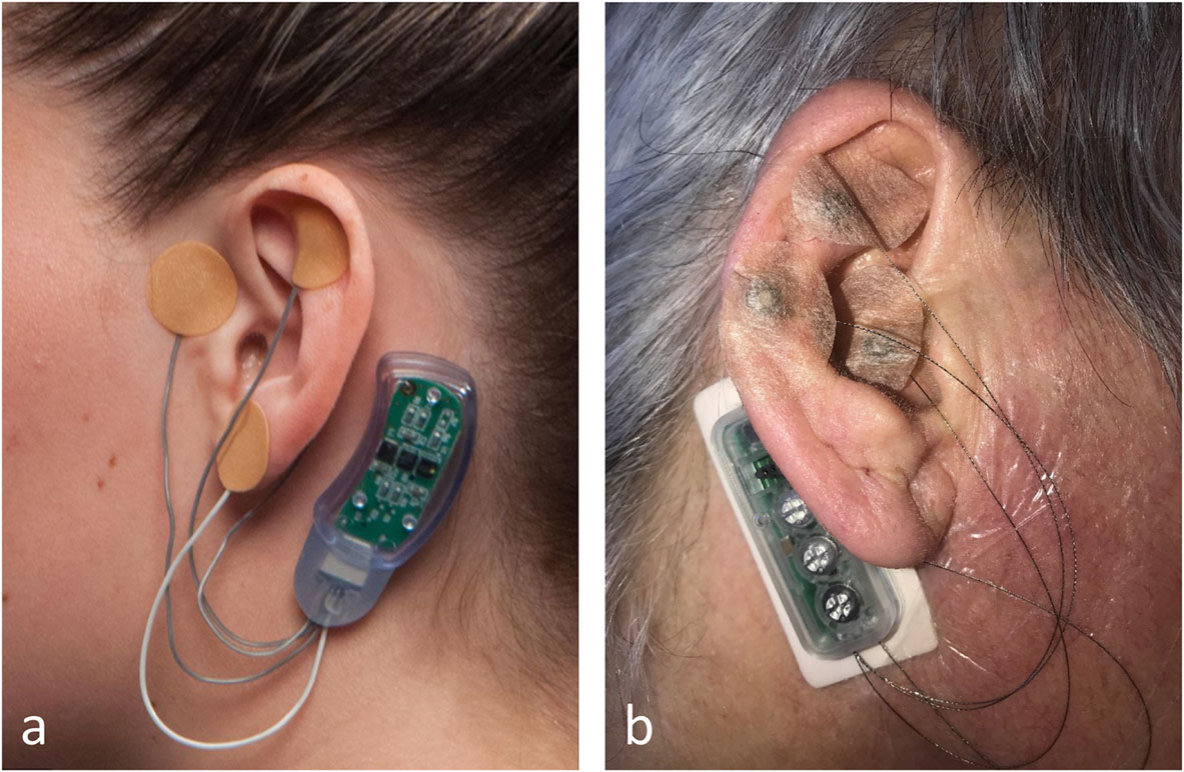
**a** The NSS-2 Bridge device placed on the ear. The body of the device is placed behind the ear. The stimulating electrodes are connected via wires and placed at key points on the ear. Each electrode contains a short penetrating needle which is used to provide percutaneous electrical stimulation. Source: Forbes.com: (source) **b** The DyAnsys Drug Relief stimulator place on the ear. The device is similar in construction to the NSS-2 Bridge device which served as the predicate for its approval. (source)

Bioelectronic medicine is an emerging field exploring neuromodulation that provides alternative, non-pharmacological treatments for various diseases (Pavlov et al. 2019). Many of these therapeutic approaches are based on vagus nerve stimulation and have already generated promising results in several chronic disorders, including rheumatoid arthritis and inflammatory bowel disease (Koopman et al. 2016; Bonaz et al. 2016; Pavlov et al. 2019; Pavlov and Tracey 2017). The therapeutic utility of non-invasive approaches, including auricular vagus nerve stimulation, is also currently being explored (Miranda and Taca 2018). Recently, the U.S. Food and Drug Administration (FDA) has approved two devices for auricular stimulation intended to treat withdrawal symptom - “NSS-2 Bridge” developed by Innovative Health Solutions (I.H.S n.d.) and “Drug Relief” aural neurostimulator mobile health device, developed by DyAnsys (DyAnsys n.d.). The “NSS-2 Bridge” consists of electrodes placed on the ear which are connected to the “hearing aid” style body of the device, which is placed behind the ear (Fig. 1a). Electrical pulses are generated at the body of the device and are delivered to the ear via the electrodes. This device is indicated for use to help in alleviating withdrawal symptoms synergizing with acute pharmacotherapy during the detoxification phase (FDA 2017). The effects of this device have been related to reduction of autonomic symptoms associated with withdrawal from opioids including sweating, gastrointestinal upset, agitation, insomnia and joint pain (FDA 2017). The vagus nerve (cranial nerve X), trigeminal (V), facial (VII), and glossopharyngeal (IX) nerves have been proposed to mediate this type of response (Deuchars et al. 2018), suggesting the activation of the parasympathetic nervous system as the core basis of the device function. It has been shown that within 20 min of activation of this device, up to 62.7% reduction of withdrawal symptoms is appreciable, while 84.6% symptoms alleviation has been observed at the end of 60 min (Miranda and Taca 2018).

Here we summarize currently available information about the use of non-invasive device-generated auricular neuromodulation in the context of alleviating symptoms of opioid withdrawal.

### Mechanisms of auricular neural stimulation for treatment of acute opioid withdrawal

The neurophysiological mechanisms underlying the beneficial effects of auricular stimulation in this context remain poorly understood. These non-invasive devices use stimulating electrodes that are attached percutaneously or transcutaneously to the distribution of peripheral afferent branches of cranial nerves. Animal studies have indicated that afferent neurons within the trigeminal, facial, glossopharyngeal and vagus nerves have projections to the brainstem, and primarily to the nucleus tractus solitarius (NTS) (Zhang and Ashwell 2001). The NTS then serves as a facilitator to relay the signals to higher brain areas including amygdala, hypothalamus and ventral medulla (Rinaman 2011; Pavlov et al. 2003). Functional MRI assessment of the effect of neural stimulation of the Cymba concha region of the external ear indicates activation of a brain network, consistent with previous findings about the brain distribution of afferent vagus nerve signaling (Frangos et al. 2015). This stimulation, compared with earlobe (control) stimulation, generates activation of the ipsilateral NTS, bilateral spinal trigeminal nucleus, dorsal raphe, locus coeruleus, and contralateral parabrachial area, amygdala, and nucleus accumbens (Frangos et al. 2015). These findings suggest possible brain mechanisms underlying anti-convulsive, antidepressant and antinociceptive effects of tVNS or electrical VNS (via implanted devices) (Frangos et al. 2015). Neuromodulation through the amygdala is related to effects on emotional lability often seen during withdrawal phase of opioid dependence (Koob and Volkow 2010). Though there is a lack of long-term findings, the existing reports on the short-term use of auricular stimulation indicate of minimal reported side effects, limited to itching and local area redness in selected cases. This makes the approach a very useful and easy to use treatment modality during the acute phase of withdrawal management.

The mechanisms of action of these types of devices involve multiple domains. The main postulated pathophysiological basis revolves around reducing the sympathetic activities while increasing parasympathetic activities (Deuchars et al. 2018). Acute taVNS in healthy volunteers has been shown to increase heart rate variability, which is indicative for an increase vagus nerve activity to the heart, and to suppress sympathetic outflow (based on microneurography data) (Clancy et al. 2014). taVNS has also been shown to stimulate efferent vagus nerve signaling to the viscera as indicated by electromyography of the stomach and increased levels of gastrin (a surrogate marker for vagus nerve activation) in 14 patients requiring open laparotomy (Hong et al. 2019). These studies indicate that tVNS alters the balance between parasympathetic and sympathetic outflow, towards parasympathetic predominance, which can be beneficial in in many conditions characterized by increased sympathetic activity. This also should improve the balance between cholinergic and adrenergic signaling with relevance to the symptomatology in opioid withdrawal (Kosten and Baxter 2019; Brown et al. 1998; Baraban et al. 1995; Cecchi et al. 2007). Neural stimulation of these nerves normalizes autonomic dysfunction, which culminates in optimization of vital signs and acts as a synergistic mechanical intervention of gastrointestinal, cardiovascular and other systems (Deuchars et al. 2018; Browning and Travagli 2014). This subsequently ameliorates physical withdrawal symptoms such as sweating, muscular and abdominal cramps, diarrhea, joint pain, tremor, anxiety and so on with less or no medications needed (Deuchars et al. 2018; Waxenbaum and Varacallo 2019).

It is important to note that the effects of auricular neural stimulation may have a much broader scope and involve other mechanisms. Of specific interest is the possible effects of this neuromodulation on immune system components. Ongoing research during the last decade has significantly advanced our understanding of the molecular mechanisms at the interface between the nervous and immune systems (Pavlov and Tracey 2017; Chavan et al. 2017), which appear to play a role in addictions. There is a bi-directional neuro-immune communication, and neural pathways regulate immune function and inflammation (Pavlov et al. 2019; Pavlov and Tracey 2017; Terrando and Pavlov 2018). Greater understanding of these mechanisms has revealed possibilities in the use of targeted neuromodulation as a therapeutic approach for disorders involving immune dysregulation and inflammation (Pavlov et al. 2019) within the emerging field of bioelectronic medicine (Pavlov et al. 2019). Targeted neuromodulation through implanted and non-invasive devices is a core aspect of bioelectronic medicine (Pavlov et al. 2019; Addorisio et al. 2019). In addition to chronic inflammatory diseases, bioelectronic medicine has the potential to provide new treatments for many disorders and neuropsychiatric conditions, including chronic migraine, epilepsy, major depressive disorder, Alzheimer’s disease, rheumatoid arthritis, pre-diabetes, paralysis, gastrointestinal diseases, and even cancer (Pavlov and Tracey 2017; Miller et al. 2019; Kaniusas et al. 2019; Eberhardson et al. 2019). Among all the available bioelectronic neuromodulatory approaches, auricular vagus nerve stimulation (aVNS) has been increasingly utilized because of its low cost, ease of application, and because it is non-invasive (Pavlov et al. 2019; Addorisio et al. 2019). The rationale for using aVNS on a specific target area of the ear (cymba concha) is based on anatomical studies suggesting that this area is the only place on the human body surface where there is afferent vagus nerve distribution (Mercante et al. 2018a; Mercante et al. 2018b). Therefore, direct stimulation of the afferent nerve fibers of the vagus nerve on the ear may be able to produce an effect similar to that produced by an implanted VNS device yet without the need for invasive surgical intervention. Evidence supporting this was recently demonstrated in a study where a form of transcutaneous auricular vagus nerve stimulation (taVNS) was applied in the treatment of rheumatoid arthritis (Addorisio et al. 2019), using mechanical stimulation of the ear. It was found that vibrotactile taVNS reduced whole-blood LPS-induced TNF production, and that the effect was sustained for a period of 24 h post stimulation. Though these findings are promising, as they indicate that taVNS can act in similar ways as VNS, an important follow up would be to combine taVNS with vagus nerve recording to better understand the neural circuits connecting the auricular branches to the cervical vagus nerve.

The effects of neural stimulation for the treatment of neuropsychiatric diseases, using taVNS or percutaneous auricular stimulation as used in the recently FDA approved devices, appear to be very similar to auricular acupuncture which has been widely used for substance use disorders (Chen et al. 2018; Baker and Chang 2016; Litscher 2019). Acupuncture has been shown to be effective for treating opioid withdrawal (Kaniusas et al. 2019; Mercante et al. 2018a; Mercante et al. 2018b; Chen et al. 2018). Studies indicate that acupuncture and electrical stimulation were more effective than medication and had fewer side effects such as insomnia, pain, and anxiety when used for treating withdrawal syndromes from acute discontinuation of opioids (Liu et al. 2009). In addition to promising clinical observations, animal studies have further explored the mechanisms of action for acupuncture in withdrawal treatment. For example, electro-acupuncture has been shown to reduce withdrawal behaviors in mice following abrupt discontinuation of morphine (Cheng et al. 1980) and to suppress naloxone-induced morphine withdrawal in rats (Fung et al. 1980). While the mechanisms of the widely used auricular acupuncture protocol are not fully understood, it is suggested that it works, at least in part, through regulation of the autonomic nervous system and immune responses (Kaniusas et al. 2019; Mercante et al. 2018b). Acupuncture needs to be administered by medical providers who have undergone specific training and special licensure, and who may be unavailable in acute detoxification units where patients receive treatment for acute opioid withdrawal. On the other hand, auricular nerve stimulation is easier to implement at these facilities.

### Transcutaneous neural stimulation as an adjunct treatment for acute opioid withdrawal

As previously mentioned, auricular stimulation of certain cranial nerves helps in ameliorating autonomic symptoms in a very short span of time. For instance, it has been reported that neurostimulation using the NSS-2 Bridge device for 30 min resulted in at least 30% alleviation of autonomic symptoms associated with opioid withdrawal (FDA 2017).

The efficacy of the NSS-2 Bridge device has been supported by a recent retrospective study with 73 patients (Miranda and Taca 2018). The device was placed at the pre-auricular region with electrodes stimulating the auricular branches of cranial nerves. Patients were assessed based on severity of Clinical Opiate Withdrawal Scale (COWS) score (Tompkins et al. 2009; Wesson and Ling 2003). The mean COWS score of 20.1 (prior to use of the device) reportedly decreases to 7.5 following 20 min of nerve stimulation, and further down to 3.1, following 60 min (Miranda and Taca 2018). This is associated with a prompt and effective amelioration of withdrawal symptoms without any additional supportive pharmacotherapeutic intervention. The device remained in place for 5 days and 64 out of 73 patients returned to the clinic on day 5 to transition to medication-assisted treatment for maintenance of their abstinence using naltrexone.

Relatively recently, in June 2018, an additional auricular stimulation device, the “Drug Relief” aural neurostimulator mobile health device, developed by DyAnsys, also received FDA 510(k) premarket approval for patients dealing with opioid addictions (Fig. 1b). This approval cited the NSS-2 Bridge (approved via the de novo review pathway in November 2017) as a predicate device. Similar to NSS-2 Bridge, the Drug Relief wearable is a transcutaneous electrical nerve field stimulator, emitting electrical pulses through tiny needles into the nerves around the ear (https://www.accessdata.fda.gov/scripts/cdrh/cfdocs/cfpmn/pmn.cfm?ID=K173861). This stimulation provides relief from symptoms associated with opioid withdrawal, including cravings, anxiety, agitation and depression. The device can be worn up to 5 days at a time, and company officials report that symptoms have eased within an hour of wearing the device.

Pain and emotional disturbances are common symptoms during opioid withdrawal. Of note, auricular neural stimulation had been used for chronic pain management with success in various health settings (e.g., reviewed in (Chen et al. 2018; Smith et al. 2018; Ju et al. 2017; Yeh et al. 2014)). Episodic migraines, chronic tension headache, severe osteoarthritis of large joints have been the prime targets for acupuncture related nerve stimulation (Baraban et al. 1995; Manheimer et al. 2018; Linde et al. 2016; MacPherson et al. 2017). The neurobiology of the auricular stimulation remains complex in chronic pain cases as it also involves modulation of the neurotransmitters and cytokines involved in pain processing and inflammation (Chavan et al. 2017; Kaniusas et al. 2019). Emotional disturbance such as major depression is a common neuropsychiatric ailment seen in clinical practice. Auricular acupuncture or neural stimulation had been utilized by practitioners with variable level of success for treatment of neuropsychiatric disorders (Smith et al. 2018; Kou et al. 2017; Shiozawa et al. 2014). This provides supporting evidence of the applicability of neural stimulation in managing multiple aspects of acute opioid withdrawal. Active ongoing research on device generated auricular neurostimulation will provide more mechanistic insight which may lead to more efficient treatment options for opioid withdrawal. In summary, non-invasive auricular neural stimulation provides a viable and efficient bioelectronic approach to treat acute opioid withdrawal in addition to currently available medications as depicted in Table 1.

## Conclusion

Experimental evidence indicates that non-invasive device-generated auricular neural stimulation of certain cranial nerves, primarily vagus nerve, provides an innovative approach to alleviate opioid withdrawal symptoms. This approach may specifically help expedite the recovery phase during acute detoxification from opioids. This can ultimately reduce the need of supportive medications and can facilitate smooth transition from detoxification to commencing medication-assisted treatment in opioid dependent patients. This treatment modality has so far been associated with only minor local side effects at the device placement site. While this non-invasive modality appears to be an exciting new opportunity in alleviating the current opioid epidemic, more studies are clearly needed to further validate the potential of this intervention and support its use with the hope of helping our patients and their families.

## Data Availability

Not applicable.

## Abbreviations

FDA: Food and Drug Administration
COWS: Clinical Opiate Withdrawal Scale
NTS: Nucleus Tractus Solitarius
VNS: Vagus Nerve Stimulation
aVNS: auricular vagus nerve stimulation
taVNS: transcutaneous auricular vagus nerve stimulation
